# Effect of Cycloplegia on the Refractive Status of Children: The Shandong Children Eye Study

**DOI:** 10.1371/journal.pone.0117482

**Published:** 2015-02-06

**Authors:** Yuan Yuan Hu, Jian Feng Wu, Tai Liang Lu, Hui Wu, Wei Sun, Xing Rong Wang, Hong Sheng Bi, Jost B. Jonas

**Affiliations:** 1 The First College of Clinical Medicine, Shandong University of Traditional Chinese Medicine, Jinan, Shandong, China; 2 Department of Ophthalmology, The Second Affiliated Hospital of Shandong University of Traditional Chinese Medicine, Jinan Shandong, China; 3 Eye Institute of Shandong University of Traditional Chinese Medicine, Jinan, Shandong, China; 4 Department of Ophthalmology, Medical Faculty Mannheim of the Ruprecht-Karls-University Heidelberg, Seegartenklinik Heidelberg, Germany; Dalhousie University, CANADA

## Abstract

**Purpose:**

To determine the effect of 1% cyclopentolate on the refractive status of children aged 4 to 18 years.

**Methods:**

Using a random cluster sampling in a cross-sectional school-based study design, children with an age of 4–18 years were selected from kindergardens, primary schools, junior and senior high schools in a rural county and a city. Auto-refractometry was performed before and after inducing cycloplegia which was achieved by 1% cyclopentolate eye drops.

**Results:**

Out of 6364 eligible children, data of 5999 (94.3%) children were included in the statistical analysis. Mean age was 10.0±3.3 years (range: 4–18 years). Mean difference between cycloplegic and non-cycloplegic refractive error (DIFF) was 0.78±0.79D (median: 0.50D; range: -1.00D to +10.75D). In univariate analysis, DIFF decreased significantly with older age (*P*<0.001;correlation coefficient r:-0.24), more hyperopic non-cycloplegic refractive error (*P*<0.001;r = 0.13) and more hyperopic cycloplegic refractive error (*P*<0.001;r = 0.49). In multivariate analysis, higher DIFF was associated with higher cycloplegic refractive error (*P*<0.001; standardized regression coefficient beta:0.50; regression coefficient B: 0.19; 95% confidence interval (CI): 0.18, 0.20), followed by lower intraocular pressure (*P*<0.001; beta: -0.06; B: -0.02; 95%CI: -0.03, -0.01), rural region of habitation (*P* = 0.001; beta: -0.04; B: -0.07; 95%CI: -0.11, -0.03), and, to a minor degree, with age (*P* = 0.006; beta: 0.04; B: 0.009; 95%CI: 0.003, 0.016). 66.4% of all eyes with non-cycloplegic myopia (≤-0.50D) remained myopic after cycloplegia while the remaining 33.6% of eyes became emmetropic (18.0%) or hyperopic (15.7%) under cycloplegia. Prevalence of emmetropia decreased from 37.5% before cycloplegia to 19.8% after cycloplegia while the remaining eyes became hyperopic under cycloplegia.

**Conclusions:**

The error committed by using non-cycloplegic versus cycloplegic refractometry in children with mid to dark-brown iris color decreased with older age, and in parallel manner, with more myopic cycloplegic refractive error. Non-cycloplegic refractometric measures lead to a misclassification of refractive error in a significant proportion of children.

## Introduction

Refractometry in individuals younger than 40 years is usually hampered by the accommodation of the lens. Depending on the age of the patient, accommodation corrects partially or fully for existing hyperopia and examination associated accommodation can additionally make the results of refractometry shift into a more myopic direction. Due to the age-dependence of the accommodative range of the lens, the influence of accommodation on refractometric results increases with younger age and it is therefore of particular concern in pediatric ophthalmology. It includes population-based studies on children [1–7]. Population-based studies on the prevalence and incidence of refractive error in school-children have often faced the problem that parents refused the participation of their children in the study if cycloplegia was applied for refractometry. To avoid a drop in the participation rate, investigators then often abstained from inducing cycloplegia and measured the refractive error using auto-refractometry under accommodating conditions. As an alternative, biometry was carried out and the ratio of corneal curvature to axial length was used as an approximated surrogate for refractive error [8,9]. It has remained unclear, whether and to which extent, refractometric results from studies avoiding cycloplegia can be compared with the findings of studies applying cycloplegia for refractometry. We conducted this study on school children to compare the results of cycloplegic refractometry with those of non-cycloplegic refractometry and to assess factors associated with the difference between both methods. The results may help to interpret the results of population-based studies on refractive error in children when cycloplegia was not applied.

## Methods

The Shandong Children Eye Study was a school-based, cross-sectional survey designed to examine the prevalence of the visual problems and to analyze risk factors of visual problems of children in the Shandong province [10,11]. Human subject research approval was obtained from the Ethics Board of the Eye Institute of the Shandong University of Traditional Chinese Medicine and the local Administration of the Education and School Board. The study was conducted according to the tenets of the Declaration of Helsinki. Purpose and methods of the study, including rare complications of cyclopentolate eye drops, were explained to the parents or guardians of children before written informed consent was obtained from the parents or guardians. The stratified cluster sampling method and the calculation of the sample size have been reported in detail previously [10,11]. In brief, according to the regional level of social and economic development, the rural county of Guanxian in Western Shandong and the relatively highly developed city of Weihai in Eastern Shandong were chosen as study sites. Stratification of clusters by grade and age ensured that children of all ages from 4 years to 18 years were representatively included into the study samples.

Eye examinations were conducted between September and December 2012 by a single trained field team that visited schools in different parts of Guanxian and Weihai. The examinations included refractometry and visual acuity measurements, assessment of stereovision and ocular motility, non-contact tonometry (Topcon CT80, Topcon Co. Tokyo, Japan), ocular biometry by laser interferometry (IOL- Master, V5.0, Carl Zeiss Meditec AG, Jena, Germany), and slit-lamp assisted biomicroscopy of the anterior and posterior segment of the eye. Using an auto-refractor (KR8900, Topcon, Itabashi, Tokyo, Japan), the refractive status was measured before and after inducing cycloplegia. According to the procedures in the manufacturer’s instruction manual, the vertex distance was 12 mm and the measurement step size was 0.25 diopters for the assessment of the spherical power and cylindrical power. Three measurements were carried out and the mean value was recorded as the final measurement. The difference between the maximum and minimum value of the measurements of spherical refractive error and cylindrical refractive error had to be less than 0.5 diopters, otherwise the measurements had to be repeated. A model eye provided by the manufacturer was used to repeatedly check the calibration of the instrument at the beginning and end of each day. Cycloplegia was induced by instilling 1% cyclopentolate eye drops (Alcon, Ft. Worth, Texas, USA) at least three times into each eye, except for eyes with diseases and except for children with an intraocular pressure higher than 25 mmHg in one or both eyes.

After reviewing for accuracy and completeness, the data was entered into the database by data administrators, applying a double entry procedure. Measurement data ranges, frequency distributions and consistency among related measurements were checked with data cleaning programs including tests for plausibility. Refractive error was expressed as the spherical equivalent, i.e. spherical refractive error plus half of the cylindrical refractive error. Myopia was defined as myopic refractive error of at least-0.50 diopters; emmetropia was defined as a refractive error of >-0.50 diopters to <0.75 diopters; hyperopia was a refractive error of 0.75 diopters or more. Within myopia, we differentiated between high myopia (myopic refractive error ≤-6.00D), moderate myopia (refractive error >-6.00 diopters and ≤-3.00 diopters), mild myopia (refractive error >-3.00 diopters and ≤ -0.50 diopters). Mild hyperopia was defined as a refractive error of ≥0.75 diopters and <3.00 diopters, moderate hyperopia as refractive error ≥3.00 diopters and <5.00 diopters, and high hyperopia as refractive error ≥5.00 diopters. The distribution of the refractive error was further analyzed by stratifying the study population by five age groups: 4 to 6 years (preschool), 7 to 9 years (grades 1–3 in primary school), 10 to 12 years (grades 4–6 in primary school), 13 to 15 years (junior high school), and 16 to18 years (senior high school).

The statistical analyses were performed using a commercially available statistical software package (SPSS for Windows, version 22.0, IBM-SPSS, Chicago, IL). The normal distribution of refractive measurements was tested with the Kolmogorov-Smirnov test. Since the conditions of a Gaussian distribution were not fulfilled (*P*<0.001), we used the Wilcoxon test to examine test differences between cycloplegic measurements and non-cycloplegic measurements. Associations between the parameters were tested, first in univariate analysis, then in a multivariate analysis. Finally, we divided the whole study population into an analysis group to form a formula to calculate the cycloplegic refractive error, and we then tested this formula in the independent test group. All *P*-values were 2-sided and were considered statistically significant when the values were less than 0.05.

## Results

Out of 6364 children invited, 6026 children (2839(47.1%) girls) were examined, representing 94.7% of those enumerated. The nonparticipants included 328 children, who refused to participate in the examination, and 10 children, who had an intraocular pressure of more than 25 mmHg in one or both eyes. All 6026 children underwent refractometry, before and after cycloplegia were induced in both eyes except for a boy with phthisis bulbi in his left eye. Among the12051 eyes of these 6026 children, 61 eyes were excluded for various reasons: the pupillary light reflex was still present after four times applying drops of 1% cyclopentolate in 54 eyes (0.45%), 3 eyes had a lens disorder, 3 eyes had a fundus disease, and one eye showed a corneal opacity. Finally, 11990 eyes of 5999 children entered the statistical analysis, with 3089 (51.5%) children from the rural region and 2910 children from the urban region. The mean age was 10. ± 3.3 years (median: 10.0 years; range: 4 to 18 years).

Mean non-cycloplegic refractive error was-0.95 ± 3.38 diopters (median: -0.50 diopters, range: -12.00 diopters to +10.00 diopters), and mean cycloplegic refractive error was-0.17 ± 2.09 diopters (median: +0.50 diopters, range: -11.75 diopters to +11.00 diopters), with a significant (*P*<0.001) difference between both values. The mean difference between the cylcoplegic refractive error and the non-cycloplegic refractive error (DIFF) was 0.78 ± 0.79 diopters (median: 0.50 diopters; range: -1.00 diopters to +10.75 diopters).

**Fig 1:**
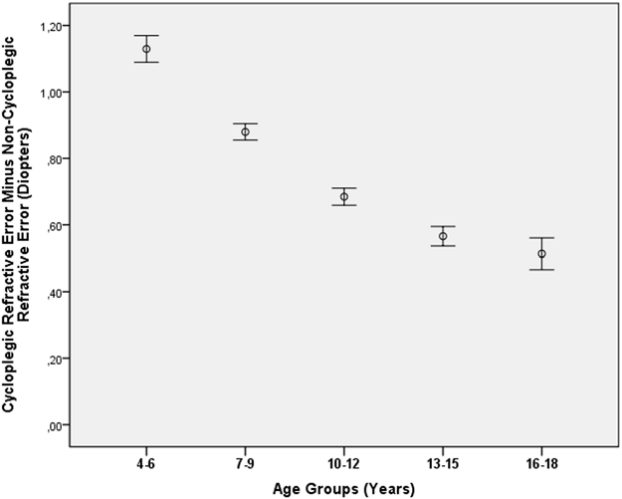
Diagram Showing the Distribution of the Difference between Cycloplegic Refractive Error and Non-Cycloplegic Refractive Error in the Shandong Children Eye Study, Stratified by Age.

**Fig 2:**
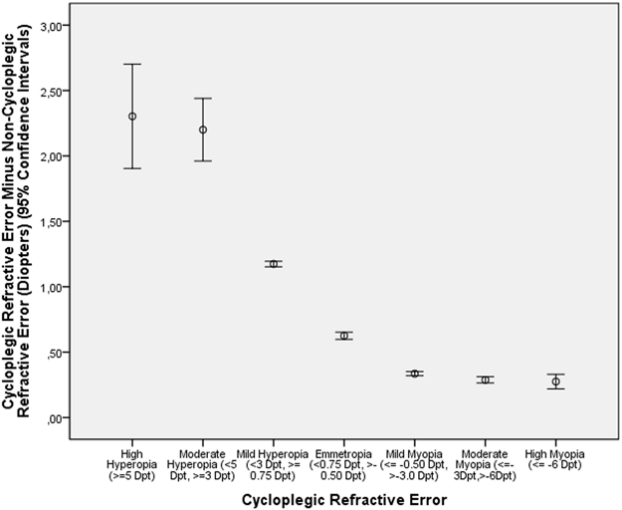
Diagram Showing the Distribution of the Difference between Cycloplegic Refractive Error and Non-Cycloplegic Refractive Error in the Shandong Children Eye Study, Stratified by Cycloplegic Refractive Error.

**Fig 3:**
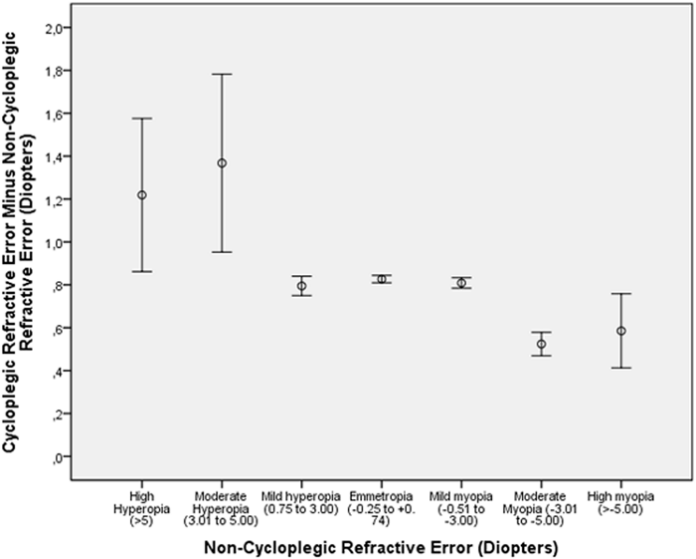
Plotting of the Difference between Cycloplegic Refractive Error minus Non-Cycloplegic Refractive Error as Compared with the Non-Cycloplegic refractive Error in the Shandong Children Eye Study.

DIFF decreased significantly (*P*<0.001) with a higher amount of myopia and with older age (Tables 1, 2) (Figs. 1–3). As a corollary, DIFF was significantly higher in the high hyperopia group and moderate hyperopia group than in the mild hyperopia group and the emmetropic group (Figs. 2–3). Eyes with high myopia (cycloplegic refractive error) and eyes with mild myopia (cycloplegic refractive error) did not differ significantly in DIFF (*P* = 0.12) nor did eyes with high myopia (cycloplegic refractive error) and eyes with moderate myopia (cycloplegic refractive error) (*P* = 0.74) (Fig. 2). In a similar manner, eyes with high myopia (non-cycloplegic refractive error) and eyes with mild myopia (non-cycloplegic refractive error) did not differ significantly in DIFF (*P* = 0.21) nor did eyes with high myopia (non-cycloplegic refractive error) and eyes with moderate myopia (non-cycloplegic refractive error) (*P* = 0.73) (Fig. 3).

**Table 1:** Prevalence of Refractive Error Before and After Cycloplegia, Stratified by Age and Gender in the Shandong Children Eye Study.

	n	Cycloplegia	≥5.00D (n (%))	≥3.00D, <5.00D (n (%))	≥0.75D, <3.00D (n (%))	>-0.50D, <0.75D (n (%))	>-3.00D, ≤ -0.50D (n (%))	>-6.00D, ≤-3.00D (n (%))	≤-6.00D (n (%))
Age (Year)									
4–6	1816	Before	10 (0.6)	6 (0.3)	501 (27.6)	1002 (55.2)	285 (15.7)	11 (0.6)	1 (0.1)
		After	19 (1.0)	56 (3.1)	1516 (83.5)	186 (10.2)	37 (2.0)	1 (0.1)	1 (0.1)
7–9	3856	Before	14 (0.4)	9 (0.2)	470 (12.2)	1973 (51.2)	1265(32.8)	114 (3.0)	11 (0.3)
		After	32 (0.8)	51 (1.3)	2236 (58.0)	854 (22.1)	598 (15.5)	78 (2.0)	7 (0.2)
10–12	3556	Before	14 (0.4)	10 (0.3)	140 (3.9)	1112 (31.3)	1760 (49.5)	477 (13.4)	43 (1.2)
		After	26 (0.7)	18 (0.5)	1079 (30.3)	854 (24.0)	1175 (33.0)	373 (10.5)	31 (0.9)
13–15	2016	Before	5 (0.2)	7 (0.3)	35 (1.7)	365 (18.1)	956 (47.4)	529 (26.2)	119 (5.9)
		After	9 (0.4)	10 (0.5)	315 (15.6)	386 (19.1)	727 (36.1)	477 (23.7)	92 (4.6)
16–18	746	Before	1 (0.1)	2 (0.3)	8 (1.1)	48 (6.4)	342 (45.8)	256 (34.3)	89 (11.9)
		After	3 (0.4)	3 (0.4)	51 (6.8)	98 (13.1)	281 (37.7)	234 (31.4)	76 (10.2)
Gender									
Boys	6328	Before	24 (0.4)	18 (0.3)	601 (9.5)	2521 (39.8)	2389 (37.8)	658 (10.4)	117 (1.8)
		After	49 (0.8)	64 (1.0)	2877 (45.5)	1304 (20.6)	1404 (22.2)	541 (8.5)	89 (1.4)
Girls	5662	Before	20 (0.4)	16 (0.3)	553 (9.8)	1979 (35.0)	2219 (39.2)	729 (12.9)	146 (2.6)
		After	40 (0.7)	74 (1.3)	2320 (41.0)	1074 (19.0)	1414 (25.0)	622 (11.0)	118 (2.1)

**Table 2:** Prevalence of Refractive Error Before and After Cycloplegia, Stratified by Region and Eye in the Shandong Children Eye Study.

	n	Cycloplegia	≥5.00D (n (%))	≥3.00D, <5.00D (n (%))	≥0.75D, <3.00D (n (%))	>-0.50D, <0.75D (n (%))	>-3.00D, ≤ -0.50D (n (%))	>-6.00D, ≤-3.00D (n (%))	≤-6.00D (n (%))
		After	40 (0.7)	74 (1.3)	2320 (41.0)	1074 (19.0)	1414 (25.0)	622 (11.0)	118 (2.1)
Region									
Rural	6176	Before	11 (0.2)	15 (0.2)	632 (10.2)	2573 (41.7)	2281 (36.9)	568 (9.2)	96 (1.6)
		After	31 (0.5)	64 (1.0)	3122 (50.6)	1192 (19.3)	1243 (20.1)	465 (7.5)	59 (1.0)
Urban	5814	Before	33 (0.6)	19 (0.3)	522 (9.0)	1927 (33.1)	2327 (40.0)	819 (14.1)	167 (2.9)
		After	58 (1.0)	74 (1.3)	2075 (35.7)	1186(20.4)	1575 (27.1)	698 (12.0)	148 (2.5)
Eye									
Right	5996	Before	21 (0.4)	15 (0.3)	528 (8.8)	2238 (37.3)	2335 (38.9)	718 (12.0)	141 (2.4)
		After	41 (0.7)	67 (1.1)	2546 (42.5)	1199 (20.0)	1444 (24.1)	593 (9.9)	106 (1.8)
Left	5994	Before	23 (0.4)	19 (0.3)	626 (10.4)	2262 (37.7)	2273 (37.9)	669 (11.2)	122 (2.0)
		After	48 (0.8)	71 (1.2)	2651 (44.2)	1179 (19.7)	1374 (22.9)	570 (9.5)	101 (1.7)
Total									
	11990	Before	44 (0.4)	34 (0.3)	1154 (9.6)	4500 (37.5)	4608 (38.4)	1387 (11.6)	263 (2.2)
		After	89 (0.7)	138 (1.2)	5197 (43.3)	2378 (19.8)	2818 (23.5)	1163 (9.7)	207 (1.7)

In univariate analysis, DIFF was significantly associated with younger age (Fig. 1) (*P*<0.001; correlation coefficient r: -0.24), female gender (*P* = 0.001; r = -0.03), rural versus urban region of habitation (*P*<0.001; r = -0.13), younger paternal age at child birth (*P*<0.001; r = -0.16), younger maternal age at child birth (*P*<0.001; r = -0.12), lower body height (*P*<0.001; r = -0.24), lighter body weight (*P*<0.001; r = -0.22), lower education of father (*P*<0.001; r = -0.07) and mother (*P*<0.001; r = -0.08), more hyperopic refractive error before cycloplegia (*P*<0.001; r = 0.13) and after cycloplegia (*P*<0.001; r = 0.49) (Fig. 2), and lower intraocular pressure (*P*<0.001; r = -0.12).

The multivariate analysis included DIFF as dependent variable and all those parameters as independent variables which were significantly associated with DIFF in the univariate analysis. In a step-wise manner, we first dropped those independent parameters for which the analysis of collinearity revealed inflation factors higher than 2: paternal age at birth (variance inflation factor: 3.7), body height (variance inflation factor: 3.7), body weight (variance inflation factor: 3.0), paternal educational level (variance inflation factor: 2.5), and non-cycloplegic refractive error (variance inflation factor: 7.3). We then dropped those independent parameters which were no longer significantly associated with DIFF: maternal educational level (*P* = 0.63), maternal age (*P* = 0.45), and gender (*P* = 0.75). DIFF finally remained to be significantly associated with higher cycloplegic refractive error (*P*<0.001), followed by lower intraocular pressure (*P*<0.001), rural region of habitation (*P* = 0.001), and, to a minor degree, with age (*P* = 0.006) (Table 3).

**Table 3:** Multivariate Analysis of the Associations of the Difference between the Cylcoplegic Refractive Error and the Non-Cycloplegic Refractive Error with Systemic Parameters and Ocular Parameters Including the Cycloplegic Refractive Error.

Parameter	*P*-Value	Standardized Correlation Beta	Regression Coefficient B	95% Confidence Interval	Variance Inflation Factor
Cycloplegic Refractive Error	<0.001	0.50	0.19	0.18, 0.20	1.53
Intraocular Pressure (mmHg)	<0.001	-0.06	-0.02	-0.03, -0.01	1.11
Area of Residence	0.001	-0.04	-0.07	-0.11, -0.03	1.17
Age (Years)	0.006	0.04	0.009	0.003, 0.016	1.52

If the cycloplegic refractive error was dropped from the multivariate analysis and the non-cycloplegic refractive error remained in the analysis, DIFF remained to be significantly associated with younger age (*P*<0.001), followed by rural region of habitation (*P*<0.001), and lower intraocular pressure (*P*<0.001), and, to a minor degree, with non-cycloplegic refractive error (*P* = 0.004) (Table 4).

**Table 4:** Multivariate Analysis of the Associations of the Difference between the Cylcoplegic Refractive Error and the Non-Cycloplegic Refractive Error with Systemic Parameters and Ocular Parameters Including the Non-Cycloplegic Refractive Error.

Parameter	*P*-Value	Standardized Correlation Beta	Regression Coefficient B	95% Confidence Interval	Variance Inflation Factor
Age (Years)	<0.001	-0.27	-0.07	-0.07, -0.06	1.44
Area of Residence	<0.001	-0.14	-0.22	-0.26, -0.18	1.15
Intraocular Pressure (mmHg)	<0.001	-0.08	-0.02	-0.03, -0.02	1.11
Non-Cycloplegic Refractive Error	0.004	-0.04	-0.02	-0.03, -0.01	1.44

Comparing the prevalence of myopia versus hyperopia in the non-cycloplegic state and the cycloplegic state revealed that only 66.4% of all eyes with a non-cycloplegic myopic refractive error (spherical equivalent ≤ -0.50 diopters) remained to be myopic under cycloplegic refractometry while the remaining 33.6% of eyes became emmetropic (18.0%) or hyperopic (15.7%) under cycloplegia (Tables 1, 2). As a corollary, the prevalence of emmetropia decreased from 37.5% before cycloplegia to 19.8% after cycloplegia while the remaining eyes became hyperopic under cycloplegia (Tables 1, 2).

## Discussion

Our cross-sectional school-based study in a rural region and in an urban region of the East Chinese province of Shandong showed that the error committed by using non-cycloplegic versus cycloplegic refractometry in children with mid to dark-brown iris color decreased with older age, and in parallel manner, with more myopic cycloplegic refractive error. One may infer that non-cycloplegic refractometric measures lead to a misclassification of refractive error in a significant proportion of children. It may be of particular importance for studies using non-cycloplegic refractometry to determine prevalences of myopia and hyperopia, and to assess associations with refractive errors, in particular myopia.

The results of our study agree with previous investigations in that non-cycloplegic refractometry appears to be too unreliable to be considered a useful method to assess the refractive error in children and in pediatric studies [1,5,7]. It agrees also with clinical experience that in young children cycloplegia is usually a must if refractometry is performed.

After adjustment for cycloplegic refractive error (in addition to intraocular pressure and age), rural region was significantly associated with a higher DIFF. The association between rural region and higher DIFF may have been explained by the finding that children in rural schools are usually more hyperopic than the children in the urban schools and therefore likely to display a greater DIFF. As shown in Table 3 and indicated by a relatively low standardized correlation coefficient of-0.04, the influence of the region of habitation on DIFF was relatively small. The comparison of the standardized correlation coefficients of the various parameters showed that by far the major factor influencing DIFF was the cycloplegic refractive error. DIFF was larger, the more hyperopic the cycloplegic refractive error was.

Due to the influence of accommodation before cycloplegia, studies avoiding cycloplegic refractometry experience a shift towards a falsely high prevalence of myopia and a falsely low prevalence of hyperopia [12,13]. The results of our study demonstrated that only 66.4% of all eyes with a non-cycloplegic myopic refractive error (spherical equivalent ≤ -0.50D) remained to be myopic under cycloplegic refractometry while the remaining 33.6% of eyes became emmetropic (18.0%) or hyperopic (15.7%) under cycloplegia. As a corollary, the prevalence of emmetropia decreased from 37.5% before cycloplegia to 19.8% after cycloplegia while the remaining eyes became hyperopic under cycloplegia.

For any comparison between non-cycloplegic refractometry and cycloplegic refractometry the device applied for auto-refractometry and method to achieve cycloplegia are important [1,14,15]. In the Shangdong Children Eye Study, we used a table-mounted auto-refractor which as compared to subjective refractometry has been shown to be more reliable to measure the refractive error in children [16]. To achieve cycloplegia, cyclopentolate 1% eye drops were applied three times per eye in our study.

It has remained unclear why in multivariate analysis, a higher DIFF was significantly associated with a lower intraocular pressure (Table 3, 4). It also remained elusive why DIFF was higher in the rural region of habitation versus the urban region.

Potential limitations of our study should be discussed. First, the data of Shandong province may be not representative for the whole of China in view of the heterogeneity of the country. Second, non-cycloplegic refractometry even if performed by an auto-refractometer, depends on the examiner instructing the child. The results of our study are therefore, as the findings of many clinical studies, depending on how carefully the examinations were conducted. Third, we have used three rounds of 1% cyclopentolate eye drops in Chinese children with irides of a mid to dark-brown color to achieve cycloplegia. Since the effect of topically applied drugs, in particular of cycloplegic drugs, depends on the iris color, the results of our study may only cautiously be transferred on populations who as compared with Chinese children have lighter irides or darker and thicker irides.

In conclusion, cycloplegia associated difference in measured refractive error increased with hyperopia in children aged >9 years and decreased with hyperopia in children aged ≤9 years, while it generally decreased with older age in children. The difference between non-cycloplegic refractive error and cycloplegic refractive error was too large to allow a prediction of cycloplegic refractive error based on non-cycloplegic refractometry. In a similar manner, the difference between cycloplegic refractive error as estimated based by age and non-cycloplegic refractive error, and cycloplegic refractive error as measured by refractometry were too large to allow a prediction of cycloplegic refractive error based on non-cycloplegic refractometry and additional data as age and gender.

## References

[1] Zhao J, Mao J, Luo R, Li F, Pokharel GP, et al (2004) Accuracy of noncycloplegic autorefraction in school-age children in China. Optom Vis Sci 81:49–55. 14747761 10.1097/00006324-200401000-00010

[2] He M, Zeng J, Liu Y, Xu J, Pokharel GP et al (2004) Refractive error and visual impairment in urban children in southern china. Invest Ophthalmol Vis Sci 45:793–799. 14985292 10.1167/iovs.03-1051

[3] He M, Huang W, Zheng Y, Huang L, Ellwein LB (2007) Refractive error and visual impairment in school children in rural southern China. Ophthalmology 114:374–382. 17123622 10.1016/j.ophtha.2006.08.020

[4] He M, Congdon N, MacKenzie G, Zeng Y, Silver JD et al (2011) The child self-refraction study results from urban Chinese children in Guangzhou. Ophthalmology 118:1162–1169. 10.1016/j.ophtha.2010.10.003 21232802 PMC6037167

[5] Hopkins S, Sampson GP, Hendicott P, Lacherez P, Wood JM (2012) Refraction in children: a comparison of two methods of accommodation control. Optom Vis Sci 89:1734–1739. 10.1097/OPX.0b013e318277182c 23142881

[6] French AN, Morgan IG, Burlutsky G, Mitchell P, Rose KA (2013) Prevalence and 5- to 6-year incidence and progression of myopia and hyperopia in Australian schoolchildren. Ophthalmology 120:1482–1491. 10.1016/j.ophtha.2012.12.018 23522969

[7] Fotedar R, Rochtchina E, Morgan I, Wang JJ, Mitchell P, et al (2007) Necessity of cycloplegia for assessing refractive error in 12-year-old children: a population-based study. Am J Ophthalmol 144:307–309. 17659966 10.1016/j.ajo.2007.03.041

[8] Grosvenor T, Scott R (1994) Role of the axial length/corneal radius ratio in determining the refractive state of the eye. Optom Vis Sci 71:573–579. 7816428 10.1097/00006324-199409000-00005

[9] Guo Y, Liu LJ, Xu L, Tang P, Lv YY, et al (2013) Myopic shift and outdoor activity among primary school children: one-year follow-up study in Beijing. PLoS One 8:e75260 10.1371/journal.pone.0075260 24086484 PMC3782472

[10] Wu JF, Bi HS, Wang SM, Hu YY, Jonas JB, et al (2013) Refractive error, visual acuity and causes of vision loss in children in Shandong, China. The Shandong Children Eye Study. PLoS One 8:e82763 10.1371/journal.pone.0082763 24376575 PMC3871613

[11] Jiang WJ, Wu JF, Hu YY, Wu H, Sun W, et al (2014) Intraocular pressure and associated factors in children: the Shandong children eye study. Invest Ophthalmol Vis Sci 55:4128–4134. 10.1167/iovs.14-14244 24876285

[12] Lan W, Zhao F, Lin L, Li Z, Zeng J, et al (2013) Refractive errors in 3–6 year-old chinese children: a very low prevalence of myopia? PLoS One 8:e78003 10.1371/journal.pone.0078003 24205064 PMC3813538

[13] Rotsos T, Grigoriou D, Kokkolaki A, Manios N (2009) A comparison of manifest refractions, cycloplegic refractions and retinoscopy on the RMA-3000 autorefractometer in children aged 3 to 15 years. Clin Ophthalmol 3:429–431. 19684866 10.2147/opth.s5145 PMC2724033

[14] Prabakaran S, Dirani M, Chia A, Gazzard G, Fan Q, et al (2009) Cycloplegic refraction in preschool children: comparisons between the hand-held autorefractor, table-mounted autorefractor and retinoscopy. Ophthalmic Physiol Opt 29:422–426. 10.1111/j.1475-1313.2008.00616.x 19523087

[15] Egashira SM, Kish LL, Twelker JD, Mutti DO, Zadnik K, et al (1993) Comparison of cyclopentolate versus tropicamide cycloplegia in children.Optom Vis Sci 70:1019–1026. 8115124 10.1097/00006324-199312000-00005

[16] Choong YF, Chen AH, Goh PP (2006) A comparison of autorefraction and subjective refraction with and without cycloplegia in primary school children. Am J Ophthalmol 142:68–74. 16815252 10.1016/j.ajo.2006.01.084

